# Molecular Characterization of Highly Pathogenic Marek’s Disease Virus Strain in Vaccinated Iraqi Pullets

**DOI:** 10.1155/av/1389742

**Published:** 2026-07-31

**Authors:** Basim Mohamed Manswr, Mohammed Abdullah Hamad, Aws El-Muntaser H. Ali, Firas R. Jameel

**Affiliations:** ^1^ Department of Pathology and Poultry Disease, College of Veterinary Medicine, University of Diyala, Baqubah, Iraq, uodiyala.edu.iq; ^2^ Department of Biotechnology, College of Applied Science, University of Fallujah, Baqubah, Iraq, uofallujah.edu.iq

**Keywords:** Gallid alphaherpesvirus 2, Iraq, Marek’s disease virus, meq gene, molecular epidemiology, poultry health, vaccine breakthrough

## Abstract

**Background:**

Marek’s disease (MD) remains a severe economic concern in poultry industry globally despite decades of vaccination strategies. Particularly challenging are viral infections due to Marek’s disease virus (MDV) (Gallid alphaherpesvirus 2, GaHV‐2) strains that can escape immune defenses in areas that do not have access to molecular surveillance data.

**Objective:**

This study performed molecular epidemiology of MDV in Iraqi commercial poultry based on extensive characterization of circulating strains, quantification of subclinical infection in vaccinated flocks, and phylogenetic sequencing to determine evolutionary relationships with local isolates.

**Materials and Methods:**

A cross‐sectional surveillance on five commercial layer pullet flocks given the CVI988 vaccine took place from March to June 2024. A combined sample of 250 birds (50 birds per flock) was collected from Alrashdia in Baghdad Province. In this study, estimates were made for samples based on 5% expected prevalence, 95% confidence level, and 3% precision. Pulpy feather follicles and tissues were isolated for molecular detection of MDV meq oncogene by PCR amplification with known primers. Sanger sequencing was performed later and phylogenetic reconstruction carried out using the maximum likelihood technique.

**Results:**

Prevalence of subclinical MDV was 3.2% (8/250) in surveyed flocks, with significant operational heterogeneity (0%–6%). One flock had an acute clinical outbreak, and 86.7% of birds afflicted during the surveillance period showed paralysis. Molecular analysis revealed genetically homogeneous strain (Iraq/Baghdad/2024) with 99.8% nucleotide identity to Iranian very virulent isolates, with clustering in the very virulent pathotype clade and high bootstrap support (98%).

**Conclusion:**

This research describes the circulation of a very virulent, genetically uniform GaHV‐2 strain capable of inducing vaccine breakthrough in Iraqi commercial poultry. The phylogenetic proximity to regional isolates suggests transboundary virus movement as a possible route of attack and highlights a gap in the level of molecular surveillance and optimization of Middle Eastern poultry production systems in immunization programs.

## 1. Introduction

Marek’s disease (MD) is one of the most economically significant neoplastic diseases of global poultry production with an annual economic burden of > 2 billion [1] worldwide. Marek’s disease virus (MDV), also known as Gallid alphaherpesvirus 2 (GaHV‐2), is linked to a complex pathophysiological syndrome of T‐cell lymphomas, progressive paralysis, immunosuppression, and loss of production [2]. The consequences of the disease are not limited to direct death but include impaired performance and increased susceptibility to secondary infections [3]. The evolution of MDV demonstrates considerable robustness, which has evolved from mild strains described in the 1960s to virulent (v), very virulent (vv), and very virulent plus (vv+) pathotypes [4]. Emerging field isolates seem to possess a high capacity for vaccine breakthrough, causing clinical disease in chickens immunized with first‐generation vaccines such as HVT (FC126) and, in instances, the bivalent version [5, 6]. As a result, molecular determinants of MDV virulence affect the meq oncogene bearing a 339‐amino acid bZIP transcription factor indispensable for transformation and latency establishment [7]. In meq, genetic polymorphisms, particularly point mutations and insertion‐deletion polymorphisms, have been associated with pathogenicity and were molecular markers to be monitored for epidemiological monitoring [8]. While pathotyping is commonly done based on proline repeat polymorphisms, 132‐bp repeat regions, or challenge studies in vaccinated birds [9], phylogenetic analysis of sequences of meq allows tracking of virus evolution and geographical extension, revealing regional clustering patterns [10]. In the Middle Eastern poultry‐production region, MD management is complicated by the limited molecular surveillance infrastructure and the cross‐border movement of birds facilitating the spread of infection [11]. On the contrary, we lack molecular evidence for circulating MDV in the Iraqi poultry industry despite a sharp increase in production [12]. As serological analyses have shown MDV was prevalent, field strain molecular diagnostics are limited [13]. Vaccinated birds can be clinically or subclinically infected with MDV; in both cases, they shed MDV from the feather follicle epithelium [14]. Since vaccines prevent clinical illness but cannot generate sterilizing immunity, such a silent circulation complicates the control of disease [15]. Subclinical infection and strain discrimination have developed to be found by modern molecular techniques, and it can provide useful surveillance systems [16]. Considering the limited knowledge on molecular epidemiology of MDV in Iraq, the following study aimed to achieve three main goals: firstly, to determine the prevalence of subclinical MDV infection in vaccinated commercial layer pullet flocks using molecular monitoring; secondly, to characterize the manifestation and molecular features of MDV strains that trigger clinical outbreaks of disease in vaccinated birds; and thirdly, to determine phylogenetic relationships between Iraqi MDV isolates and regional strains for study of evolutionary dynamics.

## 2. Materials and Methods

### 2.1. Study Design and Sampling Framework

This cross‐sectional molecular surveillance study was performed between March and June 2024 in the Alrashdia region, Baghdad Province, Iraq (33.29°N, 44.38°E). Five commercial layer pullet flocks (A–E) were enrolled, each comprising approximately 5000–7000 birds aged 8–16 weeks. All flocks had received CVI988 (Rispens) vaccine at day‐old via subcutaneous administration according to manufacturer protocols. Flocks were cared for under typical commercial practices with biosecurity in place. Sample size was determined with the formula *n* = [*Z* ^2^ × P(1−P)]/*d* ^2^, where *Z* = 1.96 (95% confidence level), *p* = 0.05 (forecast prevalence based on location), and *d* = 0.03 (precision desired). This resulted in a minimum required sample of 203 birds, and we obtained 250 samples (50 per flock) to provide enough statistical power to address sample quality concerns.

### 2.2. Sample Collection

Fifty birds were sampled systematically from each flock. The pulpy feather follicles were sampled in the femoral tract using standard protocols [17]. Individual feather samples were collected in sterile 1.5‐mL microcentrifuge tubes and transferred on ice to the laboratory within 4 h of collection. Flock B suffered an acute MD outbreak with increasing mortality during the follow‐up period (4.2% over 3 weeks). Fifteen clinically affected birds with characteristic MD signs were necropsied from this flock. Samples of liver, spleen, kidney, sciatic nerve, and bursa of Fabricius tissue were taken aseptically, placed in sterile containers, and kept at −80°C until analysis.

### 2.3. Clinical and Pathological Examination

Symptoms in affected birds were recorded in the clinical setting, including neurological (paralysis and ataxia), integumentary, and behavioral features. Comprehensive postmortem examinations were carried out on all necropsied birds in accordance with standard veterinary pathology protocols. Images of gross lesions in visceral organs, peripheral nerves, and lymphoid tissues were recorded and detailed. Histopathological examination was not performed in this study owing to resource constraints. Thus, characterization of lesions as neoplastic or inflammatory is built on gross pathological features congruent with published MD descriptions. This is a constraint to definitive lesion classification.

### 2.4. DNA Extraction

Feather follicles and tissue specimens were used to extract genomic DNA according to the manufacturer’s instructions for animal tissue, using a commercially available DNA extraction kit (GeneJET Genomic DNA Purification Kit, Thermo Fisher Scientific). In summary, ∼25 mg of tissue or 3–5 feather follicles were homogenized in 180 μL digestion buffer containing proteinase K and incubated overnight at 56°C, followed subsequently by lysis, binding, washing, and elution. DNA concentration and purity were measured using a NanoDrop spectrophotometer (Thermo Fisher Scientific), where A260/A280 ratios of 1.8 to 2.0 were deemed acceptable. DNA extracted was kept at −20°C until PCR amplification.

### 2.5. Molecular Detection and Amplification

PCR targeting the MDV meq gene was performed with 402‐bp amplified sequence primers (forward: 5′‐ATGTCTCAGGAGCCAGAGCCG‐3′; reverse: 5′‐TTATCTGCTTGCGAGGAGC‐3′) outlined by Murata et al. [18]. These primers were chosen precisely as they distinguish between v MDV field strains and the CVI988 vaccine strain. Unlike the 402‐bp product from v field strains, the CVI988 strain yields a larger meq PCR product (approximately 500 bp) due to a 59‐amino acid insertion in the transactivation domain.

PCR reactions were performed in 25 μL volumes containing 12.5 μL 2 × PCR Master Mix (Thermo Fisher Scientific), 1 μL of each primer (10 μM), 2 μL template DNA, and 8.5 μL nuclease‐free water. Thermal cycling conditions included the initial denaturation at 95°C for 5 min, followed by 35 cycles of 95°C for 30 s, 58°C for 30 s, and 72°C for 45 s, and finally extended at 72°C for 10 min. PCR products were analyzed by electrophoresis on 1.5% agarose gels stained with ethidium bromide and visualized under UV illumination. CVI988 vaccine DNA was used as a positive control to confirm primer action and the presence of vaccine virus in samples if possible.

### 2.6. DNA Sequencing and Analysis

PCR products exhibiting the anticipated 402‐bp band were purified by the PCR purification kit (GeneJET PCR Purification Kit, Thermo Fisher Scientific). Purified products were subjected to bidirectional Sanger sequencing with identical primers as used in PCR amplification. All sequences were synthesized by Macrogen Inc. (Seoul, South Korea) using an ABI 3730xl DNA analyzer (Applied Biosystems). Forward and reverse sequences were prepared and edited by BioEdit v7.2.5 software. Consensus sequences were submitted to GenBank under accession number PX495086.1.

### 2.7. Phylogenetic Analysis

Meq gene sequences were aligned in multiple sequence with MUSCLE algorithm running in MEGA X software [19]. Reference sequences of MDV with the various pathotypes and regions were obtained from GenBank (Table 1). Phylogenetic relationships were projected by maximum likelihood function following the Tamura–Nei model with gamma distribution and invariant sites. Bootstrap analysis (1000 replicates) was applied to evaluate tree topology accuracy. It should be emphasized that phylogenetic examination consisting solely on the meq gene gives an initial picture of strain association and possible virulence relationship. However, further analysis of proline repeat polymorphisms in the meq gene, assessment of 132‐bp repeat regions, or challenge studies of vaccinated birds would be required for the definitive pathotype assignment. Notably, this phylogenetic clustering suggests virulence features but is not indicative of definitive pathotype determination.

**TABLE 1:** Alignments of homology and phylogenetic analysis of the meq gene of Gallid alphaherpesvirus 2.

Strain/Isolate name	Country	GenBank accession	Identity with Iraq/Diyala/GA‐5 (%)	Classification
Iraq/Diyala/GA‐5	Iraq	PX495086.1	100.0	Local field isolate
MSBV1	Iraq	OP524128.1	98.4	Field isolate
NC	China	KM503120.1	98.4	Field isolate
SDSX03/21	China	PP967211.1	98.4	Field isolate
Yangzhou20230224	China	PX222291.1	98.4	Field isolate
HNLY101/23	China	PP967198.1	98.4	Field isolate
JiangsuTaizhou20230925	China	PX222287.1	98.4	Field isolate
EB1‐HT	Nigeria	OR592061.1	98.4	Field isolate
EB2‐FT	Nigeria	OR592062.1	98.4	Field isolate
Tetra210	Egypt	MF773445.1	98.4	Field isolate
Egypt1	Egypt	JX467678.1	98.4	Field isolate
Egypt3	Egypt	JX467680.1	98.4	Field isolate
Egypt_5	Egypt	KC161221.1	98.4	Field isolate
01	India	KT246100.1	98.4	Field isolate
ABT‐HSR‐5865	India: Haryana	JN808276.1	99.0	Field isolate
25_07_PL	Poland	HQ204792.1	98.4	Field isolate
ATE	Hungary	AY571784.1	98.4	Reference field strain
PA1	USA	MK040409.1	98.4	Reference field strain
PA5	USA	MK040413.1	98.4	Reference field strain
RB1B	Netherlands	AY571783.1	98.4	Reference virulent strain
MEQ_GIFU_1	Japan: Gifu	LC208801.1	93.8	Field isolate
HNFQ106/21	China	PP967209.1	98.4	Field isolate
CVI988 vaccine strain VS‐Meq	USA	AY243338.1	98.4	Vaccine‐associated strain
CVI988 vaccine strain S‐Meq	Japan	AB087743.1	72.4	Vaccine strain
CVI988 vaccine strain VS‐Meq	Japan	AB087744.1	72.5	Vaccine strain

### 2.8. Statistical Analysis

Prevalence estimates for the data from flocks were computed using the Wilson score. Chi‐square tests were used to compare rates of detection of MDV between flocks, with significance at *p* < 0.05. All statistical analyses were performed using R software Version 4.3.0. A limitation of this study is the small sample size, with only five flocks.

## 3. Results

### 3.1. Prevalence of Subclinical MDV Infection

Among 250 feather samples taken from the five vaccinated pullet flocks, eight were PCR positive for MDV, giving an overall prevalence of 3.2% (95% CI: 1.4%–6.2%). Detecting MDV was significantly different in flocks (*χ* ^2^ = 4.89, *p* = 0.037), from 0% in Flock C to 6.0% in Flock B (Table 2). Prevalence was greatest in Flock B, which then suffered from clinical MD outbreak in a case of infection at the subclinical level. These prevalence percentages may not hold true for all areas of Iraq.

**TABLE 2:** Distribution of subclinical GaHV‐2 infection across five commercial pullet flocks in Alrashdia, Baghdad.

Flock ID	Samples tested	Positive samples	Prevalence (%)	95% CI
A	50	1	2.0	0.1–10.9
B	50	3	6.0	1.3–16.5
C	50	0	0.0	0.0–7.1
D	50	2	4.0	0.5–13.7
E	50	2	4.0	0.5–13.7
Total	**250**	**8**	**3.2**	**1.4–6.2**

*Note:* The bold values represent the total row, summarizing the combined results across all five flocks.

### 3.2. Clinical Outbreak Characteristics

During Week 3 of the surveillance period, Flock B (12 weeks of age, 6200 birds) were acutely ill with MD‐associated clinical symptoms. The mortality rate increased from an intermediate 0.2% to 4.2% during three weeks. Of those birds, 15 had close attention to clinical and pathological features. Common clinical manifestations included paralysis, which was present in 13 birds (86.7%), either unilateral (*n* = 8) or bilateral (*n* = 5) limb paresis. Other frequent signs were depression/lethargy (60.0%), pallor of comb and wattles (46.7%), progressive weight loss (40.0%), and ataxia (33.3%). Blindness, most likely due to ocular lymphomatosis, was exhibited in two birds (13.3%) (Table 3). The whole flock was female, consistent with all commercial layer pullets at a flock.

**TABLE 3:** Clinical signs observed in 15 pullets from Marek’s disease outbreak in Flock B.

Clinical sign	Number of birds affected	Percentage (%)
Paralysis (unilateral or bilateral)	13	86.7
Depression/lethargy	9	60.0
Pallor (comb/wattles)	7	46.7
Weight loss/emaciation	6	40.0
Ataxia (incoordination)	5	33.3
Blindness	2	13.3

### 3.3. Pathological Findings

**FIGURE 1:**
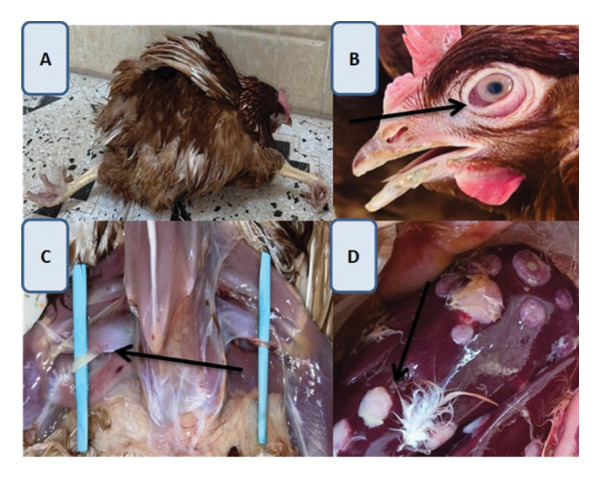
Gross pathological lesions observed in birds from Marek’s disease outbreak in Flock B. (A) Hepatomegaly with multifocal gray‐white nodular lesions (arrows) distributed throughout the parenchyma, consistent with lymphomatous infiltration. (B) Splenomegaly with similar nodular infiltrations (arrows). (C) Enlarged sciatic nerve with loss of cross‐striations (arrow), indicating peripheral nerve involvement. (D) Renal enlargement with diffuse gray discoloration and loss of normal lobulation, suggestive of lymphoid infiltration. Note: Histopathological examination was not performed; therefore, lesion characterization is based on gross morphological features consistent with published Marek’s disease descriptions.

Diagnostic tests showed that multiple organs were involved with MD. This was confirmed as systemic as well as local with all disease processes (Figure 1). Hepatomegaly with multifocal nodular lesions was noted in 11/15 birds (73.3%). Livers revealed diffuse enlargement with gray‐white foci ranging from 1 to 5 mm in diameter, distributed through the parenchyma. 9/15 birds (60.0%) had splenomegaly with similar nodular infiltrations. In 8/15 birds, the peripheral nerve, specifically the sciatic nerve, showed enlargement with loss of cross‐striations (53.3%). 7/15 birds (46.7%) had renal lesions, diffuse large enlargement with gray discoloration, and loss of normal lobulation. It was observed in 5/15 birds (33.3%) that there was bursal atrophy. Multiple organ involvement was present in multiple birds, with 6/15 (40.0%) having lesions in three or more organ systems. While the gross pathological features were compatible with lymphomatous mode of MD, lack of histopathologic confirmation precludes the final determination of these lesions as neoplastic. The nodular aspect, distribution pattern, and correlation with the clinical paralysis are almost exclusively suggestive of lymphoproliferative disease of MD, but preliminary rather than microscopic analysis.

### 3.4. Molecular Characterization

**FIGURE 2:**
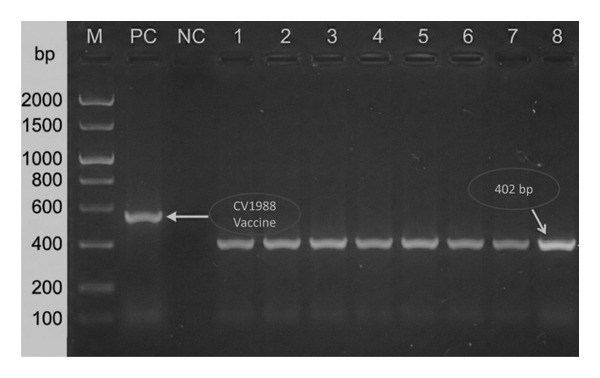
Agarose gel electrophoresis of MDV meq gene PCR products. Lanes 1–7 contain clinical isolates from Flock B. Lane 8 contains a subclinical isolate from Flock B. Lane PC (positive control) shows the CVI988 vaccine strain producing a larger amplicon (approximately more than 500 bp) due to the 59‐amino acid insertion in the transactivation domain, clearly distinguishing it from field virus isolates showing the 402‐bp product. Lane NC is the negative control. The consistent 402‐bp amplicon in field isolates indicates virulent MDV strains distinct from the vaccine virus.

PCR of MDV meq gene amplified the expected 402‐bp product in all eight subclinical infections and all 15 clinical cases, confirming the presence of MDV (Figure 2). The CVI988 vaccine control presented a larger amplicon (about 500 bp) as expected from 59 amino acid insertion in this attenuated strain, highlighting its distinctiveness from field viruses. Vaccine virus meq sequences were not produced on field samples demonstrating that circulating virus encoded field strains rather than vaccine reversion and recombination. Phylogenetic analysis of any positive samples also found the appropriate quality sequence.

### 3.5. Sequence Analysis and Phylogenetic Relationship

**FIGURE 3:**
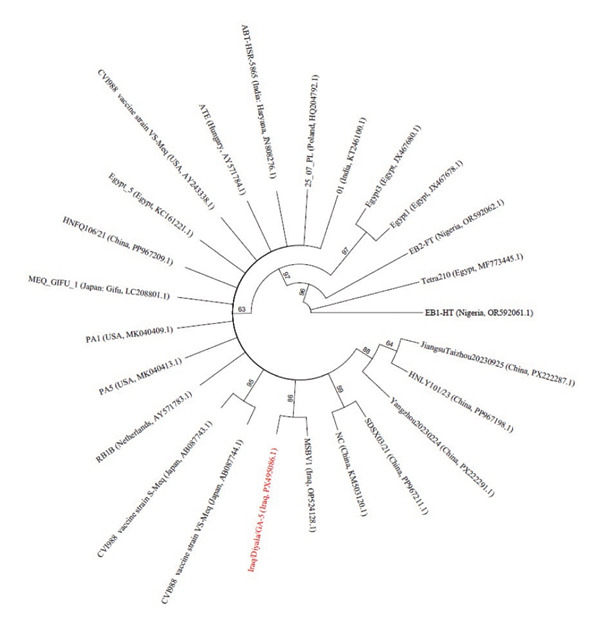
Phylogenetic tree based on partial sequences of the meq gene of Gallid alphaherpesvirus 2 (Marek’s disease virus) estimated by maximum likelihood. The Iraqi isolate Iraq/Diyala/GA‐5 (GenBank accession PX495086.1) is indicated in red and clusters with recent field isolates from different geographic locations, indicating its close genetic relationship to circulating strains. Numbers at branch nodes represent bootstrap support values with 1000 replicates. The scale bar indicates the number of nucleotide substitutions per site. Three main groups are represented: Clade I (mainly field isolates), Clade II (reference virulent strains), and Clade III (vaccine and attenuated strains including CVI988). Country of origin of sequences is given in parentheses.

The sequence analysis of the partial meq gene fragment (402 bp) indicated successful amplification in all 23 positive samples (8 subclinical and 15 clinical cases), suggesting the existence of closely related MDV strain referred to as Iraq/Diyala/GA‐5 (GenBank accession PX495086.1) (Figure 3). BLAST and pairwise identity analyses have shown high levels of nucleotide similarity with previously reported field isolates from various geographic locations, such as Iraq, China, Egypt, India, Nigeria, and Poland, with nucleotide identities from 98.4% to 99.0% (Table 1).

Phylogenetic tree construction using the maximum likelihood method and adding reference sequences from different geographic locations confirmed the clustering of the Iraqi isolate within field isolates and separation of the isolate from vaccine strains, including CVI988‐related sequences. The Iraqi isolate was closely related to field isolates from other regions and was grouped in a separate phylogenetic cluster while vaccine strains were grouped in an attenuated/vaccine‐associated cluster.

The sequence similarity of the reference vaccine strains, CVI988 S‐Meq and VS‐Meq, was significantly lower (72.4%–72.5%) than the Iraqi isolate indicating their difference from the field strains (Table 3). Although phylogenetic clustering in the vv/vv + clade and high sequence identity to verified highly v strains indicates high virulence potential for the Iraqi isolate, it is important to highlight that a clear pathotype assignment cannot be made by purely meq phylogeny. The relatively short sequenced fragment (402 bp) and the lack of functional in vivo pathogenicity study represent significant methodological limitations.

## 4. Discussion

This study is the first to have molecular characterization of MDV strains circulating in populations of poultry, overcoming a need in Middle Eastern veterinary virology. This identification of a genetically homogenous strain for vaccine breakthrough also highlights that in the face of the prevalence of vaccination, management of MD infection remains difficult. The observed 3.2% prevalence of subclinical infections in CVI988‐vaccinated flocks aligns with the prevalence reported in other regions using similar vaccination methods [20], but the direct comparison is limited because of variations in sampling techniques, flock management procedures, and vaccine administration protocols among studies.

The phylogenetic clustering of the Iraqi isolate to Iranian and Egyptian strains is consistent with the hypothesis of transboundary virus mobility via the Middle East, which might be made possible by trade in live birds and poultry commodities [21]. However, further phylogenetic analysis including other strains of the neighboring countries will be necessary for the establishment of transmission pathways and geographic distributions. Such limited reference sequences, especially from the wider Middle Eastern landscape, limit the power of phylogeographic inference.

Traditional MDV pathotyping strategies rely on functional methods such as the analysis of proline repeat polymorphisms in meq gene, analysis of 132‐bp copy numbers, or in vivo challenge studies of vaccinated chickens [22]. Although data‐based phylogeny through partial meq sequences suggests possible virulence features via clustering with known vv strains, there is no definitive pathotype assignment by sequence‐based inference. The clinical emergence in vaccinated birds and severity of pathological lesions support interpretation of high virulence, but definitive pathotyping would need additional molecular markers or experimental validation. Full meq gene sequencing, analysis of additional virulence‐related genes, and preferably controlled pathogenicity trials should be conducted in future studies to better characterize virulence. Presenting in birds, neurological manifestations (86.7% paralysis) and visceral lymphomatosis clearly illustrate the classical MD syndrome despite vaccination.

Although clinical features of lymphoproliferative disease are consistent with gross pathological features, the lack of histopathological examination was considered a methodological weakness. Additional characterization of lesion morphology and cellular composition would have been afforded with microscopical confirmation [23]. The pattern of clinical presentation and pathology is consistent with reports from other regions with the circulation of vv/vv + MDV [24, 25].

Comparison with neighboring states also shows consistent difficulties with MD control. Iranian data have documented distribution of vv MDV strains with proven ability to overcome HVT and bivalent vaccination [26], and studies carried out by other Middle East countries have also reported similar vaccine breakthrough events [27]. The genetic resemblance of genetic information among Iraqi and Iranian isolates (99.8% identity) indicates the presence of similar virus circulation patterns, or alternatively, convergent evolution under similar selective pressure.

However, available molecular data from a larger region are not available for comprehensive comparison on the same species. We identify subclinical infections in immunized birds even though vaccines have the potential to prevent disease [28]; the evidence for clinical disease of bird subclinical infections demonstrates that vaccines present on the market fail to prevent replication and shedding of the virus. This passive circulation perpetuates environmental contamination and enables evolutionary persistence of the virus under the pressure of immune selection. Improved biosecurity strategies with vaccination are also an integral part of MD control measures; in such cases, intensive cleaning methods and optimization of flock management are the complementary approaches that help to implement full‐scale MD control efforts [29].

A few limitations of the current analysis should be emphasized. First, the relatively small genomic region (402 bp of meq gene) complicates both phylogenetic resolution and virulence assessment. Other virulence markers, complete meq gene sequencing, or whole genome sequences if available may improve the molecular characterization. Then, pathotype inference based on phylogenetic clustering alone cannot be definitive since confirmation of the pathotype must rely on functional analysis on the basis of conventional markers of virulence (e.g., proline repeats and 132‐bp repeat copy numbers) either from challenge or molecular appraisal. Third, the study included only five flocks from one region, which reduces generalizability to the entire Iraqi poultry industry. Fourth, the lack of histopathological evaluation restricts the classification as definitive lesions. Fifth, the small number of individual birds limits statistical power for risk factor analysis and epidemiological interpretation. The significance of the findings in relation to the derivation of the basic molecular data of MDV circulation in Iraq and the practicality of the study at the molecular level in these locations is clear taking into account the limitations of this study.

The findings of this study emphasize a higher level of focus on surveillance of sampling in a more extensive geographical range, thorough genomic characterization, and functional pathotyping. In addition, analysis of vaccine coverage, cold chain maintenance, and time of vaccination is also required as part of the vaccination program evaluation. This regional collaborative effort to generate molecular surveillance and data sharing would enhance understanding of the epidemiology and evolution of MDV across the Middle East as well as inform local, evidence‐based control [30].

## 5. Conclusion

This study reports the circulation of a genetically homogeneous MDV strain capable of causing vaccine breakthrough in Iraqi commercial layer pullets. Molecular and phylogenetic characterization indicates that these strains can have high virulence characteristics based on clustering with previously identified vv Middle Eastern isolates but confirmation of pathotype assignment would require confirmation with functional studies. The phylogenetic correlation with regional strains is also indicative of transboundary virus spread. This highlights the importance of further molecular monitoring, comprehensive vaccination program evaluation, and regional cooperation for controlling MD in Middle Eastern poultry systems. If expanded, genomic characterization, functional pathotyping, and larger geographic sampling would enable fuller insight into MDV epidemiology in this area.

## Funding

This research received no specific grant from any funding agency in the public, commercial, or not‐for‐profit sectors.

## Conflicts of Interest

The authors declare no conflicts of interest.

## Data Availability

The raw sequence data generated in this study have been deposited in the National Center for Biotechnology Information (NCBI) and are publicly accessible. The data can be retrieved using the BioProject accession number PX495086.
